# Supplementary material for: Towards unified AI-driven fracture mechanics: the extended deep energy method (XDEM)
Source: Nat Commun. 2026 Aug 15;17:8492. doi: 10.1038/s41467-026-76748-1 (PMC13484471; doi:10.1038/s41467-026-76748-1)
Supplement: Supplementary file 3 [file 41467_2026_76748_MOESM3_ESM.zip › pic/Results/Stress_intensity_factor/Cross/XDEM/angle_60/crack_detailed_fields.pdf]

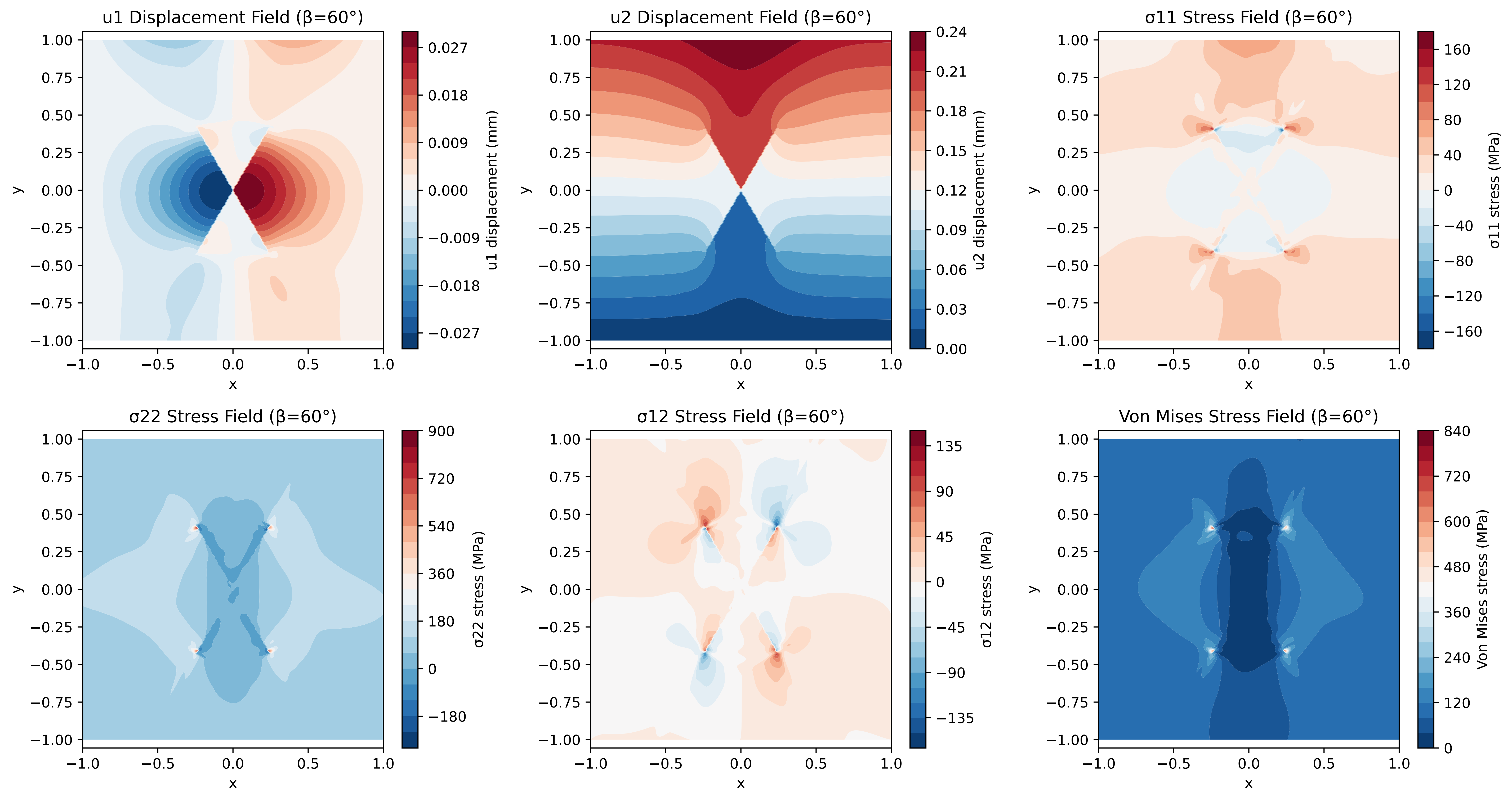

u1 Displacement Field (β=60°)
u1 displacement (mm)
0.027
0.018
0.009
0.000
−0.009
−0.018
−0.027
u2 Displacement Field (β=60°)
u2 displacement (mm)
0.24
0.21
0.18
0.15
0.12
0.09
0.06
0.03
0.00
σ11 Stress Field (β=60°)
σ11 stress (MPa)
160
120
80
40
0
−40
−80
−120
−160
σ22 Stress Field (β=60°)
σ22 stress (MPa)
900
720
540
360
180
0
−180
σ12 Stress Field (β=60°)
σ12 stress (MPa)
135
90
45
0
−45
−90
−135
Von Mises Stress Field (β=60°)
Von Mises stress (MPa)
840
720
600
480
360
240
120
0
x
y
−1.0
−0.5
0.0
0.5
1.0
1.00
0.75
0.50
0.25
0.00
−0.25
−0.50
−0.75
−1.00
